# Differential associations of transient hyperuricemia and transient hypouricemia with annual changes in estimated glomerular filtration rate in healthy participants: an observational study

**DOI:** 10.1186/s12882-026-04875-4

**Published:** 2026-03-06

**Authors:** Naoyuki Otani, Katsuyuki Tomita, Tomoaki Takata, Masanari Kuwabara, Sunao Kojima, Satoshi Miyazaki, Tetsuro Ohta, Ichiro Hisatome

**Affiliations:** 1 Department of Cardiology, Dokkyo Medical University Nikko Medical Center, 145-1 Moritomo, Nikko City, Tochigi 321-1298 Japan; 2 Department of Cardiology, NHO Yonago Medical Center, Yonago, Japan; 3 Department of Respiratory Medicine, NHO Yonago Medical Center, Yonago, Japan; 4 Division of Gastroenterology and Nephrology, Faculty of Medicine, Tottori University, Yonago, Japan; 5 Division of Public Health, Center for Community Medicine, Jichi Medical University, Shimotsuke, Japan; 6 Division of Cardiovascular Medicine, Department of Medicine, Jichi Medical University, Shimotsuke, Japan; 7 Department of Internal Medicine, Sakurajyuji Yatsushiro Rehabilitation Hospital, Yatsushiro, Japan; 8 Kumamoto University, Kumamoto, Japan; 9 Division of Cardiology, Fujii Masao Memorial Hospital, Kurayoshi, Japan; 10 Department of Cardiology, Matsue City Hospital, Matsue, Japan

**Keywords:** Dysuricemia, eGFR, CKD, Hyperuricemia, Hypouricemia, Transient

## Abstract

**Background:**

Dysuricemia, encompassing both hyperuricemia (serum uric acid: SUA > 7 mg/dl) and hypouricemia (SUA ≤ 3 mg/dL), has been linked to cardio-renal risks, but it remains unclear whether consistent or transient dysuricemia contributes to the risk of chronic kidney disease (CKD).

**Purpose:**

To investigate whether consistent and transient dysuricemia influences annual changes in the estimated glomerular filtration rate (eGFR) in healthy participants.

**Methods:**

We retrospectively analyzed 1142 healthy participants who underwent health checkups with at least four consecutive annual measurements of eGFR and SUA. Participants with consistently hyperuricemia, hypouricemia, or normouricemia (7 mg/dL ≥ SUA > 3 mg/dL) throughout follow-up were categorized as consistent hyperuricemia (*n* = 36), consistent hypouricemia (*n* = 8) and normouricemia (*n* = 759), respectively. Others were classified as transient hyperuricemia (*n* = 282) and transient hypouricemia (*n* = 57). Annualized eGFR decline was compared using ANOVA, and incident CKD stage 3 events were evaluated using χ² or Fisher’s exact tests. Multivariable analyses were performed to examine the association between baseline SUA and subsequent renal outcomes.

**Results:**

Hyperuricemia was positively associated with obesity, liver dysfunction, dyslipidemia, and higher baseline eGFR compared with normouricemia, whereas hypouricemia showed negative associations with obesity and liver dysfunction. Transient hyperuricemia was significantly associated with obesity, liver dysfunction, and dyslipidemia relative to consistent normouricemia, while transient hypouricemia was negatively associated with obesity and liver dysfunction. Transient hyperuricemia exhibited significantly faster annual eGFR decline than normouricemia, while transient hypouricemia showed no significant difference. Baseline SUA ≥ 7 mg/dL was associated with higher baseline eGFR but predicted a significantly faster subsequent eGFR decline. In multivariable linear regression, baseline SUA level was independently associated with eGFR change, whereas baseline SUA status did not independently predict incident CKD stage 3.

**Conclusion:**

Transient hyperuricemia, rather than transient hypouricemia, is associated with accelerated eGFR decline in healthy individuals. Baseline SUA provides prognostic information regarding renal function trajectory, but does not independently predict CKD stage 3, suggesting that SUA should be interpreted as a clinically useful marker of renal vulnerability rather than a proven causal factor.

**Supplementary Information:**

The online version contains supplementary material available at 10.1186/s12882-026-04875-4.

## Background

The prevalence of chronic kidney disease (CKD) has been steadily increasing in Japan, and about 13% of the Japanese adult population — approximately 13.3 million people — were predicted to have CKD [1]. Similarly, in the United States, the recent prevalence of CKD is also approximately 13% [2]. These findings highlight that CKD is a major and growing public health issue both in Japan and Western countries, particularly in aging societies. Elevated serum uric acid (SUA) levels are associated with a higher risk of both CKD incidence and onset [3, 4]. Accordingly, hyperuricemia, defined as an SUA above 7 mg/dl in Japan [5], may be considered a risk factor for CKD. The mechanisms underlying hyperuricemia-related renal dysfunction are thought to involve multiple deleterious factors, including vascular smooth muscle cell proliferation, oxidative stress, inhibition of nitric oxide production, endothelial dysfunction, and inflammation [6]. Hypouricemia, defined as an SUA below 3 mg/dl [7], has been reported to be associated with an increased risk of exercise-induced acute kidney injury and end-stage renal disease as well as urolithiasis [8]. The renal dysfunction associated with hypouricemia may be attributable to the loss of urate’s antioxidant capacity [9].

Recently, the concept of *dysuricemia*, which encompasses the risks of both hyperuricemia and hypouricemia in relation to organ damage, has been proposed from a pathophysiological perspective [10]. Treatments with urate-lowering agents or ascorbic acid as an antioxidant have been suggested to mitigate the risks of dysuricemia based on the pathophysiology of uric acid. However, recent studies have shown that uric acid-lowering therapy for hyperuricemia in patients with preexisting CKD does not improve kidney outcomes [11, 12], and no significant difference in annual eGFR decline was observed between participants with hypouricemia and those with normouricemia [7], raising questions about dysuricemia-induced kidney dysfunction. These inconsistent findings may be partly explained by transient fluctuations in SUA levels.

SUA levels exhibit diurnal and seasonal variations [13, 14]. Nevertheless, most traditional studies have simply analyzed the relationship between baseline SUA levels and longitudinal changes in eGFR [15]. Therefore, it is important to address the effects of changes in SUA levels on concurrent changes in eGFR during follow-up. Dysuricemia can be classified into consistent and transient types [8, 16], but few studies have evaluated the effects of these categories on changes in eGFR over time. In the present study, we investigated whether consistent and transient dysuricemia influences annual changes in eGFR among healthy participants.

## Methods

### Study design and study population

**Fig. 1:**
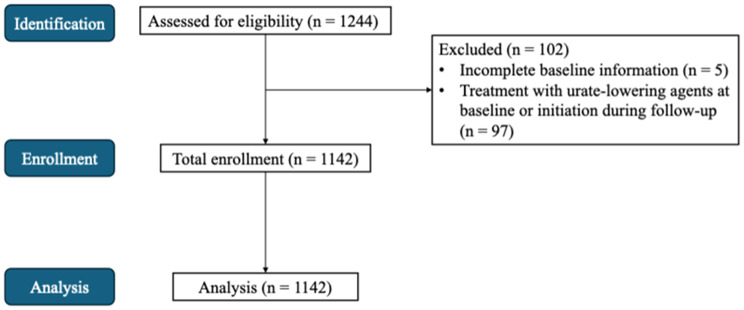
Flowchart of participant inclusion and exclusion. Abbreviations: SUA, serum uric acid; eGFR, estimated glomerular filtration rate

The study was a retrospective observational cohort study using repeated annual health checkup data collected in April 2014 and March 2024. All healthy participants were examined in the medical checkup center, NHO Yonago Medical Center, and had examinations at least once a year with a median follow-up of 5 (range: 4–10) years. All blood samples were collected in the morning after an overnight fast during annual medical check-ups conducted at approximately the same time each year. A total of 1244 participants whose eGFR and serum uric acid (SUA) had been measured for at least four years were initially assessed for eligibility. Of these, 102 participants were excluded due to incomplete baseline information or the use of urate-lowering agents either at baseline or during the follow-up period, resulting in 1142 participants enrolled in the study. All enrolled participants were included in the subsequent analyses (Fig. 1). Hyperuricemia is defined as an SUA above 7 mg/dl and hypouricemia is defined as an SUA below 3 mg/dl, thus, normouricemia is defined as an SUA between 3 mg/dl and 7 mg/dl. The participants whose SUA levels consistently exceed 7 mg/dl or below 3 mg/dl during the observational periods were defined as consistent hyperuricemia (*n* = 36) and hypouricemia (*n* = 8) participants and participants whose SUA levels are consistently between 3 mg/dl and 7 mg/dl during the observational periods were defined as consistent normouricemia (*n* = 759), while others were defined as transient hyperuricemia (*n* = 282) or transient hypouricemia (*n* = 57). Baseline patient data, including age, sex, body mass index (BMI), incidence of complications such as hypertension (HT), diabetes, dyslipidemia (DL), and medications, were obtained from medical records. Data were analyzed using an autoanalyzer. SUA, serum creatinine (Scr) levels were measured using the uricase- peroxidase (POD) method to evaluate uric acid metabolism. eGFR for the Japanese population was calculated using the following equation: eGFR (mL/min/1.73 m^2^) = 194 × serum Cr-1.094 × age-0.287 (for females) × 0.739, where Cr = serum creatinine level (mg/dL). The eGFR and SUA levels were measured over 4 consecutive years. Incident CKD stage 3 was defined as an eGFR < 60 mL/min/1.73 m^2^ at final follow-up among participants with baseline eGFR ≥ 60 mL/min/1.73 m^2^. No participants progressed to CKD stage 4 or 5 during the follow-up period.

The Institutional Review Boards of the NHO Yonago Medical Center approved the study protocol (approval numbers: 0406 − 11). Informed consent was obtained in the form of opt-out. Patient privacy was protected in accordance with the Declaration of Helsinki.

### Slope analysis with Bayesian regression model

To estimate the slope of association between ΔSUA and ΔeGFR, a linear regression with a Bayesian regression model (brms) was performed according to that previously reported [17]. The formula is represented as annual eGFR decline = b0 + bi * (annual SUA decline), b0: intercept, bi: coefficient. Regarding bi, b1 implies the slope of association between annual SUA decline and annual eGFR decline in consistent hyperuricemia, b2 in transient hyperuricemia, b3 in normal uricemia, b4 in transient hypouricemia, and b5 in permanent hypouricemia. The sampling algorithm used is the No U-Turn Sampler (NUTS), which is one of the gradient-based Markov chain Monte Carlo (MCMC) algorithms. The convergence of each MCMC was assessed graphically with trace plots and with the Gelman-Rubin Rhat convergence diagnostics [18]. A Rhat of less than 1.1 is considered evidence that chains have converged. MCMC methods were used with four chains, 500 burn-in iterations, and 6,000 iterations for generating samples of posterior distributions.　The Bayesian analysis was conducted using the brms package in the R Project for Statistics Computing version 3.2.5 (Vienna, Austria) [19].

### Alluvial (Sankey) flow diagrams

Alluvial (Sankey) flow diagrams [20] are a data visualization technique that emphasizes flow/movement/change from one state to another or one time to another. We used the alluvial flow diagrams to visualize the CKD risk of participants over time to observe the annual eGFR change. We created an alluvial flow diagram using R software version 4.1.0.

### Statistical analysis for multiple comparison

Differences among groups were analyzed using R version 4.4.2 (R Foundation for Statistical Computing, Vienna, Austria) and SPSS Statistics version 30.0 (IBM Corp., Armonk, NY, USA). Kruskal–Wallis tests were used for overall group comparisons. One-way ANOVA was applied for comparisons of annualized eGFR slope when normality assumptions were met. Categorical variables were compared using χ² tests or Fisher’s exact tests as appropriate. Univariable and multivariable logistic regression analyses were performed to evaluate factors associated with incident CKD stage 3. Odds ratios with 95% confidence intervals were calculated using logistic regression models or 2 × 2 contingency tables, as appropriate. The Benjamini–Hochberg procedure was applied to correct for multiple comparisons [21]. A two-sided p value < 0.05 was considered statistically significant.

## Results

### Differences in demographic data among hyperuricemia, normouricemia, and hypouricemia participants

Supplemental Table 1 summarizes the demographic differences among hyperuricemia, normouricemia, and hypouricemia participants. Compared with normouricemia, participants with hyperuricemia had a significantly higher proportion of males, body mass index (BMI), prevalence of hypertension, and smoking, as well as higher aspartate aminotransferase (AST), alanine aminotransferase (ALT), and triglyceride (TG) levels, whereas high-density lipoprotein (HDL) cholesterol was significantly lower. In hypouricemia participants, the proportion of females was higher; however, BMI, prevalence of hypertension and levels of ALT, AST, and TG were significantly lower than those in both hyperuricemia and normouricemia participants. Both annual SUA slope and eGFR slope differed significantly between the hyperuricemia and normouricemia participants, with a steeper eGFR decline observed in participants with hyperuricemia.

### Differences in demographic data among transient hyperuricemia, consistent normouricemia, and transient hypouricemia participants

Dysuricemia encompasses both hyperuricemia and hypouricemia, and can be classified into transient or consistent types. Based on these criteria, participants were categorized into five groups: consistent hyperuricemia, transient hyperuricemia, consistent normouricemia, transient hypouricemia, and consistent hypouricemia (Table 1). We compared demographic data of transient hyperuricemia and transient hypouricemia participants with those of consistent normouricemia. In transient hyperuricemia participants, BMI and prevalence of hypertension were significantly higher, whereas these parameters were significantly lower in transient hypouricemia participants compared with consistent normouricemia. Similarly, ALT, AST, and TG levels were significantly higher in transient hyperuricemia but significantly lower in transient hypouricemia compared with consistent normouricemia. HDL cholesterol was significantly lower in transient hyperuricemia compared with consistent normouricemia. Both the uric acid slope and the eGFR slope differed significantly between transient hyperuricemia and consistent normouricemia participants, with a steeper eGFR decline observed in transient hyperuricemia.

**Table 1:**
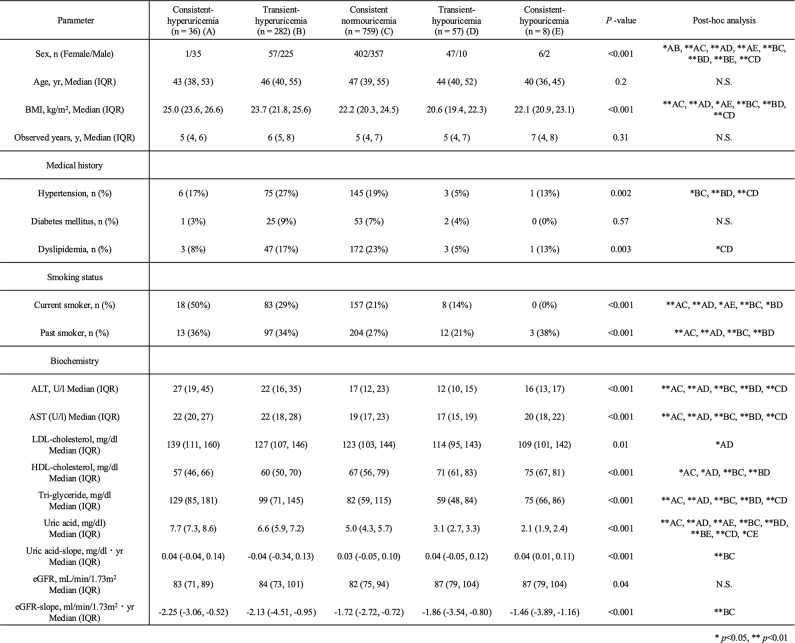
Demographic data of the eligible participants categorized by serum uric acid levels and fluctuated status

### The slope of association between ΔSUA and ΔeGFR in transient and consistent dysuricemia

**Fig. 2:**
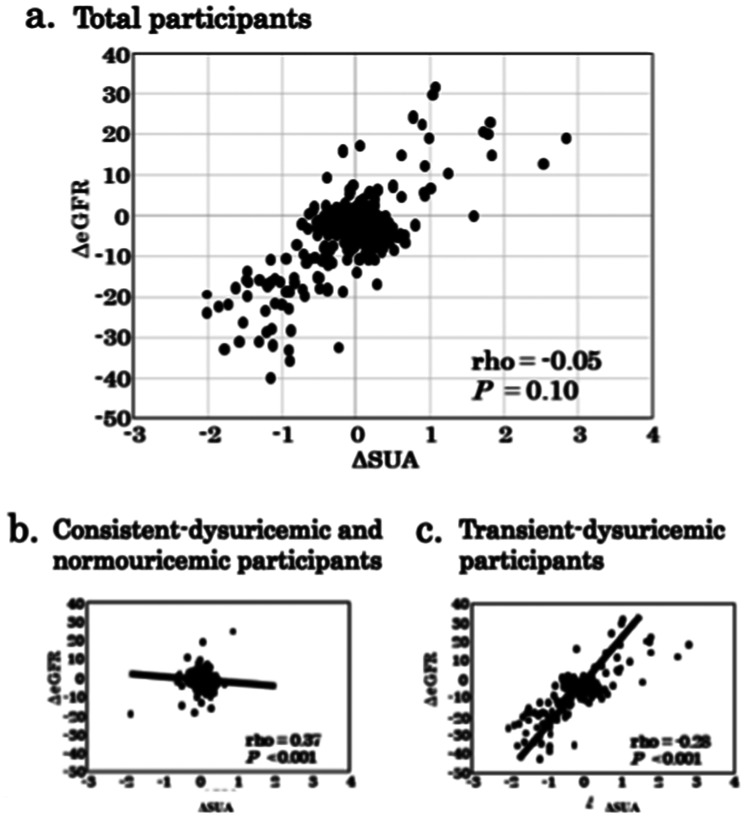
Correlation of annual change in estimated glomerular filtration rate (ΔeGFR) with annual change in serum uric acid (ΔSUA) in total participants (**a**), consistent-dysuricemia and normouricemic participants (**b**), and transient-dysuricemia participants (**c**). Consistent hyperuricemia is defined as sustained SUA during the observed periods on hyperuricemia (SUA > 7 mg/dL). Transient hyperuricemia is defined as fluctuating SUA, shuttling between hyperuricemia (SUA > 7 mg/dL) and normouricemia (7 mg/dL ≥ SUA > 3 mg/dL) during the observed periods. Consistent normouricemia is defined as sustained SUA during the observed periods on normouricemia (7 mg/dL ≥ SUA > 3 mg/dL). Transient hypouricemia is defined as fluctuated SUA, to shuttle between hypouricemia (SUA ≤ 3 mg/dL) and normouricemia (7 mg/dL ≥ SUA > 3 mg/dL) during the observed periods. Consistent hypouricemia is defined as sustained SUA during the observed period on hypouricemia (SUA ≤ 3 mg/dL)

Supplemental Figure S1 shows Kernel density estimates and simulated trace in the Bayesian linear model of selected parameters with the slope of association between ΔSUA and ΔeGFR calculated and interactions associated with consistent-hyperuricemic participants (a), transient-hyperuricemic participants (b), consistent-normouricemic participants (c), transient-hypouricemic participants (d) and consistent-hypouricemic participants (e) using the sampling algorithm of NUTS, which correctly predict the trend of population depicted by posterior distribution through prior distribution and likelihood. Supplemental Figure S2 illustrates the slope of association between ΔSUA and ΔeGFR in total cohort and according to each uric acid status group. Dysuricemia includes both hyperuricemia and hypouricemia. Supplementary Figure S3 shows Kernel density estimates and simulated trace in the Bayesian linear model of selected parameters with the slope of association between ΔSUA and ΔeGFR calculated and interactions associated with total participants (a), consistent-dysuricemia and normouricemic participants (b), and transient-dysuricemia participants (c) using the sampling algorithm of NUTS. A Rhat of < 1.1 is considered evidence of adequate chain convergence across all posterior distributions (Table 2). Figure 2 shows the slope of association between ΔSUA and ΔeGFR in total participants (panel a), in consistent dysuricemia and normouricemia (panel b), and in transient dysuricemia (panel c). A significant negative correlation was observed in the slope of association between ΔSUA and ΔeGFR of consistent dysuricemia and normouricemia (panel b), whereas a significant positive correlation was found in the slope of transient dysuricemia participants (panel c). To further evaluate potential heterogeneity within transient hyperuricemia, particularly with respect to sex-related differences in serum uric acid trajectories, sex-stratified sensitivity analyses were performed. In transient hyperuricemia, the slope of the association between ΔSUA and ΔeGFR was positive in both males (β = 11.50, 95% CI: −6.06 to 29.06) and females (β = 13.04, 95% CI: −4.43 to 30.50). In contrast, slopes in consistent normouricemia were close to zero in both males (β = −4.04, 95% CI: −37.34 to 29.26) and females (β = −0.28, 95% CI: −36.89 to 36.33) (Supplementary Table S2).

**Table 2:** Regression coefficient from Bayesian linear regression model on the slope of association between annual SUA decline and annual eGFR decline in three groups: total participants, consistent dysuricemic and normouricemic participants and transient dysuricemic participants

	Regression coefficient			Rhat	Bulk_ESS	Tail_ESS
	Est.	Est. Error	95%CI			
(A) Total participants						
Intercept	-2.21	0.12	-2.45 – -1.97	1.00	4379	2866
Slope	10.58	0.35	9.83–11.23	1.00	3209	3039
(B) Consistent dysuricemic and normouricemic participants						
Intercept	-1.72	0.10	-1.90 – -1.53	1.00	3900	3011
Slope	-0.14	0.57	-1.27–0.97	1.00	4364	3044
(C) Transient dysuricemic participants						
Intercept	-2.64	0.31	-3.25 – -2.06	1.00	4034	3145
Slope	12.38	0.54	11.30–13.43	1.00	3868	2667

Abbreviations: Bulk_ESS, bulk effective sample size; CI, credible interval; Est., estimate; Tail_ESS, tail effective sample size

For each parameter, Bulk_ESS and Tail_ESS are effective sample size measures, and Rhat is the potential scale reduction factor for the splint chains

### Annual eGFR decline and CKD stage progression according to transient hyperuricemia, normouricemia, and transient hypouricemia

To address potential heterogeneity among dysuricemia phenotypes, we compared annualized eGFR decline (eGFR slope) across the three groups (Supplementary Table S3). Mean eGFR slope was − 0.83 ± 2.22 mL/min/1.73 m²/year in the transient hyperuricemia group, − 0.30 ± 0.61 in the normouricemia group, and − 0.31 ± 1.58 in the transient hypouricemia group. There was a significant overall difference among groups (one-way ANOVA, *p* < 0.001). In pairwise comparisons with the normouricemia group as the reference, transient hyperuricemia was associated with significantly greater eGFR decline (*p* < 0.001), whereas transient hypouricemia showed no significant difference from normouricemia (*p* = 0.98). We also evaluated incident CKD stage 3 events as a categorical outcome (Supplementary Table S3). The incidence of CKD stage 3 was 13.5% (38/282) in the transient hyperuricemia group, 6.5% (49/759) in the normouricemia group, and 10.5% (6/57) in the transient hypouricemia group. Pairwise comparisons with the normouricemia group as the reference were performed using χ² or Fisher’s exact tests as appropriate. Transient hyperuricemia was associated with a significantly higher incidence of CKD stage 3 events compared with normouricemia (unadjusted odds ratio 2.09, 95% CI 1.40–3.12, *p* < 0.001), whereas transient hypouricemia showed no significant difference from normouricemia (odds ratio 1.71, 95% CI 0.70–4.17, *p* = 0.27). Because these comparisons were unadjusted and did not account for differences in baseline characteristics or follow-up duration, the findings were interpreted as descriptive associations rather than evidence of causality.

### Transition of CKD risk in transient and consistent dysuricemia

**Fig. 3:**
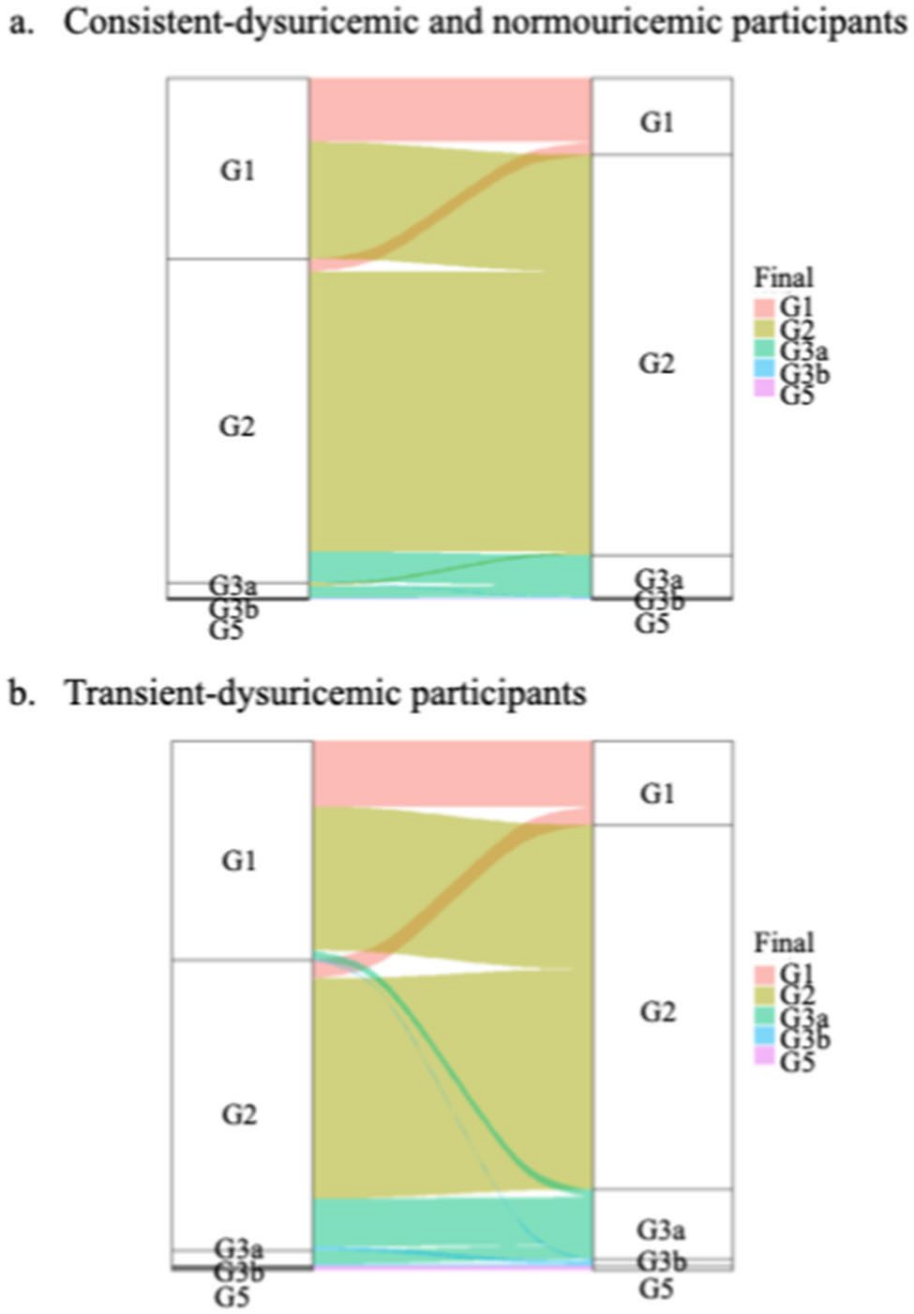
Transition of chronic kidney disease (CKD) risk in transient and consistent dysuricemia. Alluvial flow diagrams depicting CKD risk over time by visualizing estimated glomerular filtration rate (eGFR) staging at initial and final observed points in consistent-dysuricemia and normouricemia participants (**a**), and transient-dysuricemia participants (**b**). Consistent hyperuricemia is defined as sustained SUA during the observed periods on hyperuricemia (SUA > 7 mg/dL). Transient hyperuricemia is defined as fluctuated SUA, to shuttle between hyperuricemia (SUA > 7 mg/dL) and normouricemia (7 mg/dL ≥ SUA > 3 mg/dL) during the observed periods. Consistent normouricemia is defined as sustained SUA during the observed periods on normouricemia (7 mg/dL ≥ SUA > 3 mg/dL). Transient hypouricemia is defined as fluctuated SUA, to shuttle between hypouricemia (SUA ≤ 3 mg/dL) and normouricemia (7 mg/dL ≥ SUA > 3 mg/dL) during the observed periods. Consistent hypouricemia is defined as sustained SUA during the observed period on hypouricemia (SUA ≤ 3 mg/dL)

Figure 3 presents alluvial flow diagrams depicting CKD risk over time. The diagrams indicate the transition of participants who experienced an annual eGFR decline from stage G1 and G2 to stage G3a and G3b in consistent dysuricemia and normouricemia (panel a) and in transient dysuricemia (panel b). The transition of CKD stage from G1 and G2 to G3a and G3b was higher in transient dysuricemia participants (12.0%) compared with that of consistent dysuricemia and normouricemia participants (6.2%).

### Multivariable analysis for CKD stage 3 development

To determine whether baseline SUA independently predicts progression to CKD stage 3, we performed multivariable logistic regression analysis restricted to participants with preserved renal function at baseline (baseline eGFR ≥ 60 mL/min/1.73 m^2^; *n* = 269, events = 38). In this model, age (adjusted OR 1.067, 95% CI 1.024–1.112, *p* = 0.002), baseline eGFR (adjusted OR 0.951, 95% CI 0.924–0.979, *p* < 0.001), and follow-up duration (adjusted OR 1.253, 95% CI 1.046–1.501, *p* = 0.014) were significant predictors of CKD stage 3 at final follow-up. Male sex was associated with a lower risk of CKD progression (adjusted OR 0.372, 95% CI 0.134–1.027, *p* = 0.056). Importantly, baseline SUA status (baseline SUA > 7 vs. ≤ 7 mg/dL) was not an independent predictor of CKD stage 3 (adjusted OR 1.354, 95% CI 0.597–3.074, *p* = 0.468) (Table 3).

**Table 3:**
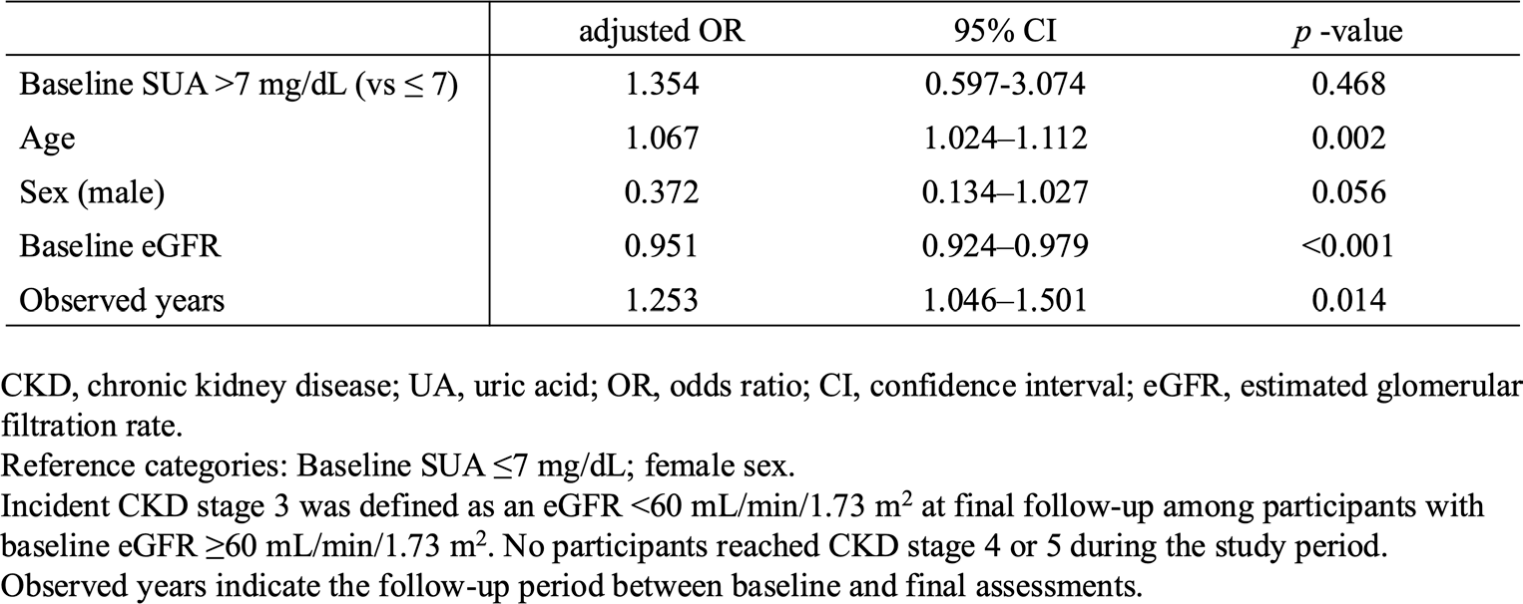
Multivariable logistic regression for CKD stage 3 at final follow-up (N=269, events=38)

### Baseline serum uric acid and subsequent renal function trajectory

To evaluate the prognostic significance of baseline serum uric acid (SUA) within individuals with transient hyperuricemia and transient hypouricemia, we examined the association between initial SUA level and subsequent changes in renal function. In univariate comparisons, participants with baseline SUA > 7 mg/dL had significantly higher baseline eGFR than those with SUA ≤ 7 mg/dL (112.98 ± 44.29 vs. 83.95 ± 14.68 mL/min/1.73 m^2^, *p* = 2.27 × 10^− 10^). Despite better initial renal function, the baseline SUA > 7 mg/dL group exhibited a markedly faster decline in renal function (eGFR slope − 1.81 ± 2.56 vs. − 0.04 ± 1.58 mL/min/1.73 m^2^/year, *p* = 9.62 × 10^− 11^). The incidence of CKD stage 3 did not differ significantly between groups (11.3% vs. 16.6%, *p* = 0.226). In multivariable linear regression adjusted for age, sex, baseline eGFR, and follow-up duration, baseline SUA group (> 7 vs. ≤ 7 mg/dL) was independently associated with subsequent eGFR change (standardized β = −0.11, *p* = 0.016). In a separate model in which baseline SUA was analyzed as a continuous variable, baseline SUA (per mg/dL) was also independently associated with eGFR change (standardized β = −0.17, *p* = 0.011) (Table 4).

**Table 4:**
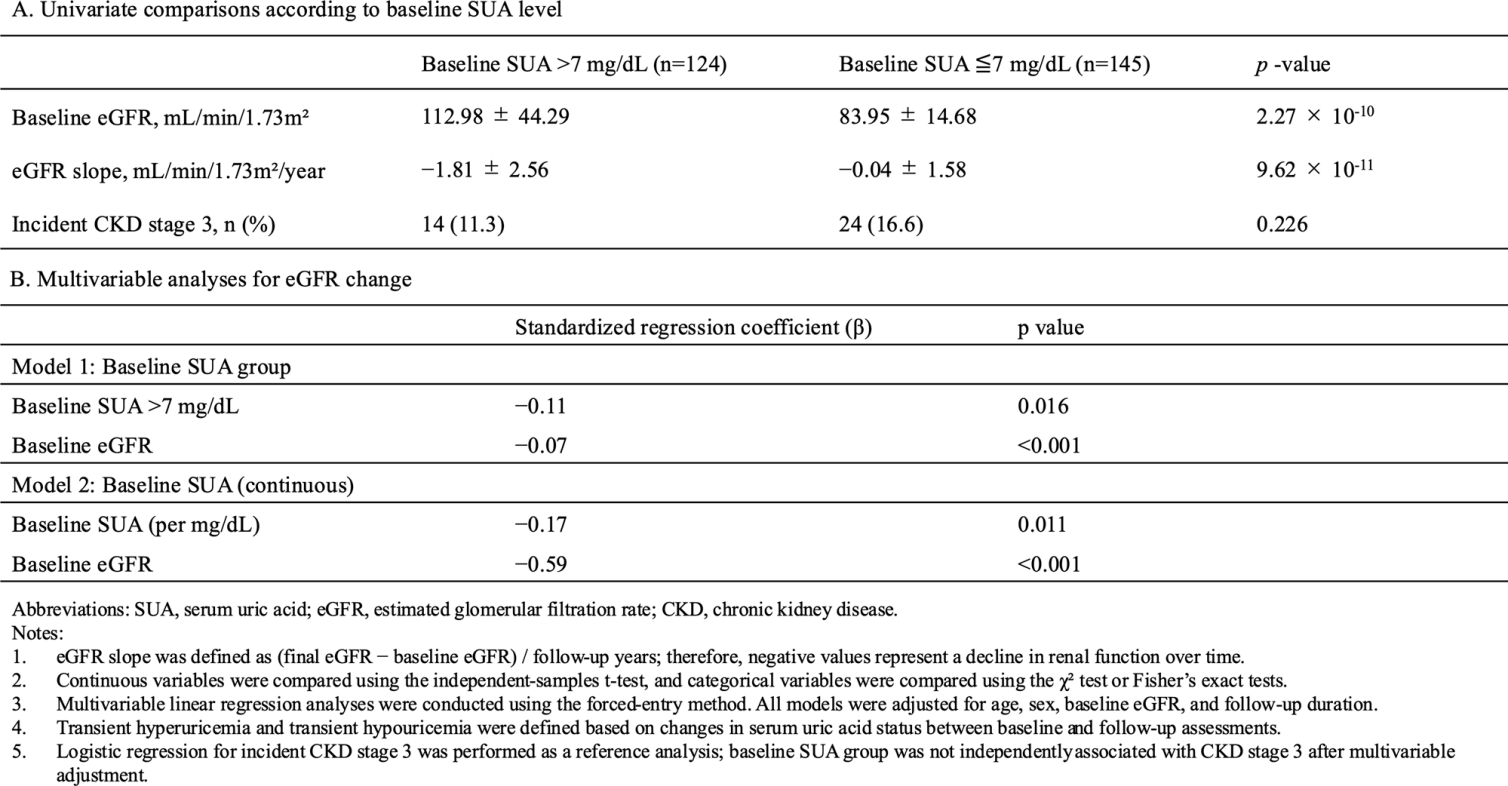
Association of baseline serum uric acid and subsequent renal outcomes in individuals with transient hyperuricemia and transient hypouricemia

## Discussion

In this study, we demonstrated that: (1) hyperuricemia was positively associated with high BMI, hypertension, and liver dysfunction compared with normouricemia, whereas hypouricemia was negatively associated with obesity, hypertension, and liver dysfunction; (2) high BMI, hypertension, and liver dysfunction were significantly enriched in transient hyperuricemia participants, but negatively enriched in transient hypouricemia participants compared with consistent normouricemia; (3) ΔSUA was positively correlated with ΔeGFR in transient dysuricemia participants, while ΔSUA was negatively correlated with ΔeGFR in consistent dysuricemia and normouricemia participants and (4) transient hyperuricemia, but not transient hypouricemia, was associated with significantly greater annual eGFR decline compared with normouricemia. Furthermore, transient hyperuricemia was associated with a significantly higher incidence of CKD stage 3 events, whereas transient hypouricemia showed no significant difference from normouricemia.

Interestingly, high BMI, hypertension, and liver dysfunction were positively enriched in transient hyperuricemia participants but negatively enriched in transient hypouricemia participants with consistent normouricemia as the reference. These findings suggest that changes in SUA levels during follow-up may be related to lifestyle-related diseases [22]. This is consistent with the concept that hyperuricemia can serve as a marker associated with lifestyle-related diseases associated with insulin resistance, since SUA levels are proportional to serum insulin levels [23]. Notably, not only consistent hyperuricemia, but also transient hyperuricemia were significantly associated with lifestyle-related metabolic status. Although the underlying mechanisms remain unclear, the demographic differences between consistent and transient hyperuricemia suggest that sex-related differences play a role. Indeed, hyperuricemia has been reported to increase the risk of renal and cardiovascular disease in women rather than in men [24, 25]. As transient hyperuricemia in women might be included in the study population of the previous reports, transient hyperuricemia may enhance the risk of lifestyle-related diseases particularly in females.

Dysuricemia has been proposed as a novel concept encompassing the kidney disease risk of both hyperuricemia and hypouricemia [10]. However, evidence regarding its risk remains controversial. Several clinical trials have suggested that pharmacological reduction of SUA levels can improve kidney function in CKD patients [26–28], but recent studies have failed to demonstrate the beneficial effects of urate-lowering therapy on CKD incidence or progression [29]. In both the randomized controlled CKD-FIX trial of patients with stage 3–4 CKD [11] and the PERL trial of patients with long-standing type 1 diabetes and mild-to-moderate CKD [12], treatment with allopurinol did not attenuate the annual decline in eGFR. Moreover, no significant difference in the annual decline of eGFR was observed among renal hypouricemia patients and normouricemia participants [7]. This uncertainty may arise from the conventional approach of assessing only the relationship between baseline SUA levels and longitudinal changes in eGFR. Dysuricemia defined by a single baseline measurement may include both consistent and transient dysuricemia [16]. Therefore, it is essential to evaluate the effects of changes in SUA levels on concurrent changes in eGFR during follow-up. In the present study, ΔSUA was positively correlated with ΔeGFR in transient dysuricemia, whereas it was negatively correlated with ΔeGFR in consistent dysuricemia and normouricemia, which is in line with previous reports [30–33]. In addition, analyses focusing on baseline SUA demonstrated that individuals with baseline SUA ≥ 7 mg/dL had higher baseline eGFR yet experienced significantly faster subsequent eGFR decline. Multivariable models confirmed that baseline SUA level was independently associated with eGFR change after adjustment for age, sex, baseline eGFR, and follow-up duration. These findings support the interpretation that baseline SUA provides prognostic information regarding future renal function trajectory. However, baseline SUA did not independently predict incident CKD stage 3 events, indicating that the present data are insufficient to establish SUA as a causal determinant of CKD progression. Rather, SUA should be interpreted primarily as a clinically useful marker of renal vulnerability. Since uric acid itself acts as a danger signal in acute renal ischemia, transient hyperuricemia may represent a danger signal potentially associated with subsequent eGFR decline [34]. It is well recognized that soluble uric acid is a potent scavenger of peroxynitrite [35, 36]. If so, the transient hyperuricemia might reflect a physiological response to increased oxidative stress within the kidney. However, in the present study, transient hyperuricemia was consistently associated with faster eGFR decline and a higher incidence of CKD stage progression, and therefore our findings do not support a clearly protective role of transient hyperuricemia on renal function. Rather than representing a compensatory or protective response, transient hyperuricemia may serve as a marker of underlying metabolic or hemodynamic instability that predisposes individuals to subsequent renal vulnerability. Regardless of the precise mechanisms on the relationship between transient dysuricemia and eGFR decline, transient hyperuricemia may partly explain the inconsistency of the results of clinical trials using uric acid-lowering agents (ULAs) on kidney function in CKD patients with hyperuricemia. Many clinical studies have enrolled hyperuricemic patients at baseline, likely including cases of transient hyperuricemia. Thus, reductions in SUA levels by ULAs may also be contaminated with natural reductions of SUA levels occurring in transient hyperuricemia, and effects of reduction of SUA levels on eGFR in the participants with transient hyperuricemia may counteract the effects of reduced SUA by ULAs on eGFR. From this perspective, this study included participants undergoing routine health checkups without strict eligibility criteria, which may have allowed us to capture the natural course of transient uric acid abnormalities in the general population. Consequently, our findings may help bridge the gap between tightly controlled clinical trials and everyday clinical practice, emphasizing that short-term fluctuations in SUA levels are not merely random variation but may represent important determinants of renal function trajectories.

There are several limitations in the present study. As an observational study, the findings demonstrate associations but not causation. In addition, comparisons of incident CKD stage 3 events were based on unadjusted analyses and may have been influenced by differences in baseline characteristics and follow-up duration. Indeed, it has been reported that higher variability in SUA is independently associated with the risk of kidney impairment [37], and that declines in both SUA and eGFR were significantly greater in groups with higher SUA [4, 38], which is consistent with some of our observations. However, in the Coronary Artery Risk Development in Young Adults Study, changes in serum creatinine were strongly positively associated with rising SUA levels in relatively young adults [39]. Another retrospective study found that elevated baseline SUA and increases in SUA over time were independent risk factors for rapid eGFR decline over five years [4, 33, 40]. These discrepancies highlight the need for further investigation. The classification of dysuricemia patterns depends heavily on measurement intervals and follow-up duration. Given the well-known diurnal and seasonal variability of serum urate, potential misclassification bias cannot be excluded. Because our analysis was based on real-world health checkup data rather than a strictly controlled cohort, unmeasured confounders and background heterogeneity could not be fully eliminated.

## Conclusion

Transient dysuricemia, particularly transient hyperuricemia, may serve as a clinically useful marker for accelerated annual eGFR decline in healthy participants.

## Supplementary Information

Below is the link to the electronic supplementary material.

Supplementary Material 1

Supplementary Material 2

Supplementary Material 3

Supplementary Material 4

Supplementary Material 5

Supplementary Material 6

Supplementary Material 7

## Data Availability

The data underlying this article will not be made publicly available because of the privacy of the study participants. These data will be shared upon reasonable request to the corresponding author.

## Abbreviations

SUA: Serum uric acid
CKD: Chronic kidney disease
eGFR: Estimated glomerular filtration rate
BMI: Body mass index
HT: Hypertension
DL: Dyslipidemia
Scr: Serum creatinine
POD: Peroxidase
NUTS: No U-Turn Sampler
MCMC: Markov chain Monte Carlo
AST: Aspartate aminotransferase
ALT: Alanine aminotransferase
TG: Triglyceride
HDL: High-density lipoprotein
