# Supplementary material for: Differential associations of transient hyperuricemia and transient hypouricemia with annual changes in estimated glomerular filtration rate in healthy participants: an observational study
Source: BMC Nephrol. 2026 Mar 6;27:236. doi: 10.1186/s12882-026-04875-4 (PMC13077997; doi:10.1186/s12882-026-04875-4)

Supplementary Figure S2

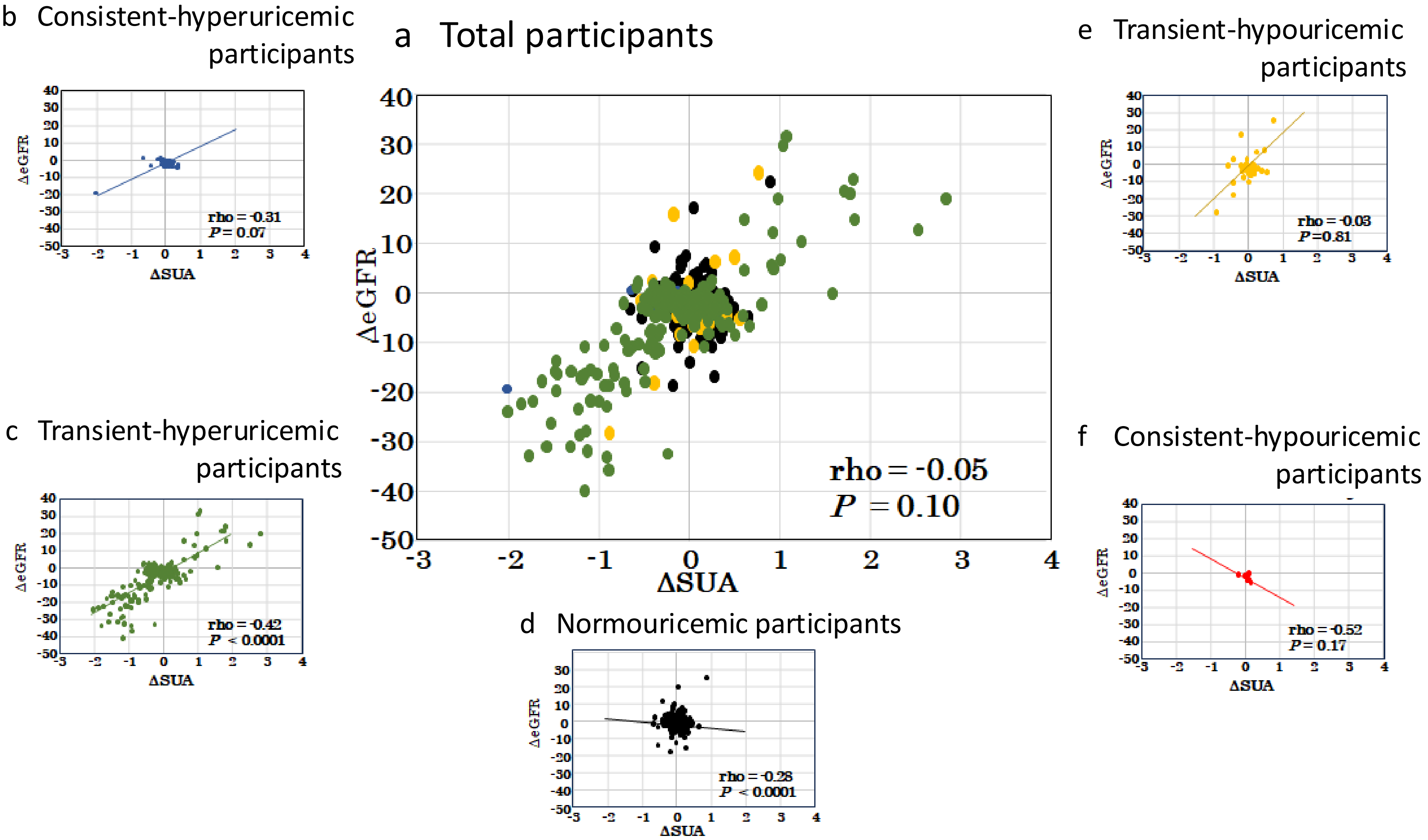

Supplement: Supplementary file 2 — Supplementary Material 2 [file 12882_2026_4875_MOESM2_ESM.pdf]
