# Supplementary material for: Differential associations of transient hyperuricemia and transient hypouricemia with annual changes in estimated glomerular filtration rate in healthy participants: an observational study
Source: BMC Nephrol. 2026 Mar 6;27:236. doi: 10.1186/s12882-026-04875-4 (PMC13077997; doi:10.1186/s12882-026-04875-4)
Supplement: Supplementary file 4 — Supplementary Material 4 [file 12882_2026_4875_MOESM4_ESM.pdf]

Supplemental Table S1

	Hyperuricemia (n = 318) (A)	Normouricemia (n = 759) (B)	Hypouricemia (n = 65) (C)	*P* -value	Post-hoc analysis
Sex, n (Female/Male)	58/260	402/357	53/12	<0.001	**AB, **AC, **BC
Age, y, Median (IQR)	45 (39, 54)	47 (39, 55)	44 (40, 52)	0.24	N.S.
BMI, kg/m^2, Median (IQR)	23.9 (22.1, 25.8)	22.2 (20.3, 24.5)	20.7 (19.5, 22.4)	<0.001	**AB, **AC, **BC
Observed years, y, Median (IQR)	5 (5, 8)	5 (4, 7)	5 (4, 7)	0.47	N.S.
Medical history					
Hypertension, n (%)	81 (25%)	145 (19%)	4 (6%)	<0.001	*AB, *AC, **BC
Diabetes mellitus, n (%)	26 (8%)	53 (7%)	2 (3%)	0.37	N.S.
Dyslipidemia, n (%)	50 (16%)	172 (23%)	4 (6%)	<0.001	*AB, **AC, **BC
Smoking status					
Current smoker, n (%)	101 (32%)	157 (21%)	8 (12%)	<0.001	**AB, **AC; compared with past smoker
Past smoker, n (%)	110 (35%)	204 (27%)	15 (23%)		**AB, **AC; compared with non-smoker
Biochemistry					
ALT, U/l, Median (IQR)	22 (16, 35)	17 (12, 23)	13 (10, 16)	<0.001	**AB, **AC, **BC
AST, U/l, Median (IQR)	22 (18.3, 28)	19 (17, 23)	17 (15, 20)	<0.001	**AB, **AC, **BC,
LDL-cholesterol, mg/dl, Median (IQR)	127 (109, 147)	123 (103, 144)	114 (95, 143)	0.01	*AC
HDL-cholesterol, mg/dl, Median (IQR)	60 (49, 70)	67 (56, 79)	71 (61, 83)	<0.001	**AB, **AC
Tri-glyceride, mg/dl, Median (IQR)	103 (72, 145)	82 (59, 115)	64 (49, 84)	<0.001	**AB, **AC, **BC
Uric acid, mg/dl, Median (IQR)	6.8 (6.0, 7.4)	5 (4.3, 5.7)	2.9 (1.6, 3.3)	<0.001	**AB, **AC, **BC
Consistent/transient, n	36/282	759/0	8/57		
Uric acid-slope, mg/dl・yr, Median (IQR)	-0.03 (-0.28, 0.13)	0.03 (-0.05, 0.10)	0.04 (-0.04, 0.11)	<0.001	**AB, *AC
eGFR, ml/min/1.73m^2, Median (IQR)	84 (73, 98.8)	83 (75, 94)	87 (79, 103)	0.05	N.S.
eGFR slope, ml/min/1.73m^2・yr, Median (IQR)	-2.15 (-4.12, -0.91)	-1.72 (-2.72, -0.72)	-1.80 (-3.57, -0.80)	<0.001	**AB

* $p<0.05$, ** $p<0.01$
