# Supplementary material for: Differential associations of transient hyperuricemia and transient hypouricemia with annual changes in estimated glomerular filtration rate in healthy participants: an observational study
Source: BMC Nephrol. 2026 Mar 6;27:236. doi: 10.1186/s12882-026-04875-4 (PMC13077997; doi:10.1186/s12882-026-04875-4)
Supplement: Supplementary file 5 — Supplementary Material 5 [file 12882_2026_4875_MOESM5_ESM.pdf]

Supplemental Table 2. Sex-stratified sensitivity analysis of the association between annual changes in serum uric acid (ΔSUA) and estimated glomerular filtration rate (ΔeGFR)

Parameter	Transient-hyperuricemia			Consistent normouricemia		
	All	Male	Female	All	Male	Female
Number, n	282	225	57	759	357	402
Δ Uric acid-slope, mg/dl・yr Median (IQR)	-0.04 (-0.34, 0.13)	-0.03 (-0.22, 0.12)	-0.31 (-0.74, 0.19)	0.03 (-0.05, 0.10)	0.02 (-0.06, 0.10)	0.03 (-0.04, 0.11)
Δ eGFR-slope, ml/min/1.73m^2・yr Median (IQR)	-2.13 (-4.51, -0.95)	-1.95 (-3.40, 0.90)	-4.77 (-19.16, 1.66)	-1.72 (-2.72, -0.72)	-1.56 (-2.67, -0.60)	-1.82 (-2.83, -0.80)
Slope β (ΔeGFR per ΔSUA)	12.28	11.50	13.04	-1.91	−4.04	−0.28
Standard Error	9.30	8.96	8.91	18.06	16.99	18.68
95% Confidence Interval	-5.95 to 30.50	−6.06 to 29.06	−4.43 to 30.50	-37.31 to 33.48	−37.34 to 29.26	−36.89 to 36.33

Abbreviations: ΔSUA, annual change in serum uric acid; ΔeGFR, annual change in estimated glomerular filtration rate.
Sex-stratified linear regression analyses were performed to evaluate the association between ΔSUA and ΔeGFR separately in transient hyperuricemia and consistent normouricemia. Positive β values indicate that greater decreases in SUA were associated with greater declines in eGFR. Analyses were conducted using simple linear regression models with ΔeGFR as the dependent variable and ΔSUA as the independent variable within each sex and group.
