# Supplementary material for: Differential associations of transient hyperuricemia and transient hypouricemia with annual changes in estimated glomerular filtration rate in healthy participants: an observational study
Source: BMC Nephrol. 2026 Mar 6;27:236. doi: 10.1186/s12882-026-04875-4 (PMC13077997; doi:10.1186/s12882-026-04875-4)
Supplement: Supplementary file 6 — Supplementary Material 6 [file 12882_2026_4875_MOESM6_ESM.pdf]

Supplementary Table S3. Comparison of incident CKD stage 3 events among normouricemia, transient hyperuricemia, and transient hypouricemia groups

	N	eGFR slope (mL/min/1.73 m^2/year) Mean ± SD	P-value (vs Consistent normouricemia)*	CKD stage 3 events n (%)	OR (vs Consistent normouricemia)	95% CI	*P* –value**
Consistent normouricemia	759	−0.30 ± 0.61	Reference	49 (6.5%)	Reference	–	–
Transient-hyperuricemia	282	−0.83 ± 2.22	<0.001	38 (13.5%)	2.09	1.40–3.12	<0.001
Transient-hypouricemia	57	−0.31 ± 1.58	0.98	6 (10.5%)	1.71	0.70–4.17	0.27

Abbreviations: eGFR, estimated glomerular filtration rate; CKD, chronic kidney disease; OR, odds ratio; CI, confidence interval.

* Annualized eGFR slope (continuous variable) was compared using one-way analysis of variance (ANOVA), followed by pairwise comparisons versus the normouricemia group.

** Incident CKD stage 3 events were compared with the normouricemia group using χ^2 test or Fisher's exact test as appropriate. Unadjusted odds ratios (ORs) and 95% confidence intervals (CIs) were calculated from 2 × 2 contingency tables.

Incident CKD stage 3 was defined as an eGFR <60 mL/min/1.73 m^2 at follow-up among participants without CKD stage 3 at baseline.

P-values represent pairwise comparisons of each group versus the normouricemia group.
