# Supplementary material for: Differential associations of transient hyperuricemia and transient hypouricemia with annual changes in estimated glomerular filtration rate in healthy participants: an observational study
Source: BMC Nephrol. 2026 Mar 6;27:236. doi: 10.1186/s12882-026-04875-4 (PMC13077997; doi:10.1186/s12882-026-04875-4)
Supplement: Supplementary file 7 — Supplementary Material 7 [file 12882_2026_4875_MOESM7_ESM.docx]

**Supplementary Table S1**

Demographic characteristics of participants with hyperuricemia, normouricemia, and hypouricemia at the initial assessment.

Abbreviations: IQR, interquartile range; BMI, body mass index; AST, aspartate aminotransferase; ALT, alanine aminotransferase; LDL, low density lipoprotein: HDL, high density lipoprotein; eGFR, estimated glomerular filtration rate.

**Supplementary Table S2**

Sex-stratified sensitivity analysis of the association between annual changes in serum uric acid (ΔSUA) and estimated glomerular filtration rate (ΔeGFR).

Abbreviations: ΔSUA, annual change in serum uric acid; ΔeGFR, annual change in estimated glomerular filtration rate.

Sex-stratified linear regression analyses were performed to evaluate the association between ΔSUA and ΔeGFR separately in transient hyperuricemia and consistent normouricemia. Positive β values indicate that greater decreases in SUA are associated with greater declines in eGFR. Analyses were conducted using simple linear regression models with ΔeGFR as the dependent variable and ΔSUA as the independent variable within each sex and group.

**Supplementary Table S3**

Comparison of incident CKD stage 3 events among normouricemia, transient hyperuricemia, and transient hypouricemia groups

Abbreviations: eGFR, estimated glomerular filtration rate; CKD, chronic kidney disease; OR, odds ratio; CI, confidence interval.

* Annualized eGFR slope (continuous variable) was compared using one-way analysis of variance (ANOVA), followed by pairwise comparisons versus the normouricemia group.

** Incident CKD stage 3 events were compared with the normouricemia group using χ² test or Fisher’s exact test as appropriate. Unadjusted odds ratios (ORs) and 95% confidence intervals (CIs) were calculated from 2 × 2 contingency tables.

Incident CKD stage 3 was defined as an eGFR <60 mL/min/1.73 m² at follow-up among participants without CKD stage 3 at baseline.

P-values represent pairwise comparisons of each group versus the normouricemia group.

**Supplementary Figure S1**

Kernel density estimates and trace plots from Bayesian linear models illustrating the posterior distributions of the slope of the association between annual changes in estimated glomerular filtration rate (ΔeGFR) and serum uric acid (ΔSUA) for consistent hyperuricemic (a), transient hyperuricemic (b), normouricemic (c), transient hypouricemic (d), and consistent hypouricemic (e) participants. Posterior sampling was performed using the No-U-Turn Sampler (NUTS).

Consistent hyperuricemia was defined as sustained serum uric acid (SUA) >7 mg/dL during the observation period. Transient hyperuricemia was defined as fluctuation between hyperuricemia (SUA >7 mg/dL) and normouricemia (7 mg/dL ≥ SUA >3 mg/dL). Consistent normouricemia was defined as sustained normouricemia (7 mg/dL ≥ SUA >3 mg/dL). Transient hypouricemia was defined as fluctuation between hypouricemia (SUA ≤3 mg/dL) and normouricemia. Consistent hypouricemia was defined as sustained hypouricemia (SUA ≤3 mg/dL).

**Supplementary Figure S2**

Correlation between annual changes in estimated glomerular filtration rate (ΔeGFR) and serum uric acid (ΔSUA) in total participants (a), consistent hyperuricemic (b), transient hyperuricemic (c), normouricemic (d), transient hypouricemic (e), and consistent hypouricemic (f) participants.

Consistent hyperuricemia was defined as sustained serum uric acid (SUA) >7 mg/dL during the observation period. Transient hyperuricemia was defined as fluctuation between hyperuricemia (SUA >7 mg/dL) and normouricemia (7 mg/dL ≥ SUA >3 mg/dL). Consistent normouricemia was defined as sustained normouricemia (7 mg/dL ≥ SUA >3 mg/dL). Transient hypouricemia was defined as fluctuation between hypouricemia (SUA ≤3 mg/dL) and normouricemia. Consistent hypouricemia was defined as sustained hypouricemia (SUA ≤3 mg/dL).

**Supplementary Figure S3**

Kernel density estimates and simulated trace plots from the robust Bayesian linear regression model for the slope of annual changes in estimated glomerular filtration rate per annual change in serum uric acid (ΔeGFR/ΔSUA) in total participants (a), consistent-dysuricemia and normouricemia participants (b), and transient-dysuricemia participants (c), generated using the No U-Turn Sampler (NUTS).

Consistent hyperuricemia was defined as sustained serum uric acid (SUA) >7 mg/dL during the observation period. Transient hyperuricemia was defined as fluctuation between hyperuricemia (SUA >7 mg/dL) and normouricemia (7 mg/dL ≥ SUA >3 mg/dL). Consistent normouricemia was defined as sustained normouricemia (7 mg/dL ≥ SUA >3 mg/dL). Transient hypouricemia was defined as fluctuation between hypouricemia (SUA ≤3 mg/dL) and normouricemia. Consistent hypouricemia was defined as sustained hypouricemia (SUA ≤3 mg/dL).
